# Should the COVID-19 lockdown be relaxed or intensified in case a vaccine becomes available?

**DOI:** 10.1371/journal.pone.0273557

**Published:** 2022-09-02

**Authors:** Alessandra Buratto, Maddalena Muttoni, Stefan Wrzaczek, Michael Freiberger

**Affiliations:** 1 Dipartimento di Matematica Tullio Levi-Civita, Università degli Studi di Padova, Padova, Italy; 2 International Institute for Applied Systems Analysis (IIASA), Laxenburg, Austria; 3 Wittgenstein Centre (IIASA, OeAW, University of Vienna), Vienna Institute of Demography (VID), Vienna, Austria; Education University of Hong Kong, CHINA

## Abstract

Immediately after the start of the COVID-19 pandemic in Early 2020, most affected countries reacted with strict lockdown to limit the spread of the virus. Since that time, the measures were adapted on a short time basis according to certain numbers (i.e., number of infected, utilization of intensive care units). Implementing a long-term optimal strategy was not possible since a forecast when R&D will succeed in developing an effective vaccination was not available. Our paper closes this gap by assuming a stochastic arrival rate of the COVID-19 vaccine with the corresponding change in the optimal policy regarding the accompanying optimal lockdown measures. The first finding is that the lockdown should be intensified after the vaccine approval if the pace of the vaccination campaign is rather slow. Secondly, the anticipation of the vaccination arrival also leads to a stricter lockdown in the period without vaccination. For both findings, an intuitive explanation is offered.

## Introduction

Since the beginning of 2020, the COVID-19 pandemic has kept the world in suspense. In addition to distance rules and hygiene measures, the immediate reaction of most countries was a lockdown of all nonessential parts of the economy. Although these measures were quite successful in terms of reducing infections and saving lives (e.g., [1] estimates that 3.1 million deaths have been averted by lockdown measures in 11 countries in Europe), the economy suffered enormously (according to [2] the decrease of the world output fell up to 23% in spring 2020), and it became clear quite soon that an efficient vaccination will be the only viable option to end the pandemic in the long term.

Since then, all over the world additional research effort was put into exploring COVID-19 with significant resources allocated towards the development of vaccines and medications. Initially, it was speculated that vaccines could become available about 1.5 years after the beginning of the pandemic. However, due to the large efforts and unprecedented international collaboration, several vaccinations with different technologies (vector vaccine, mRNA, dead vaccine, etc.) have been developed in record breaking time (the vector vaccine “Convidicea”, for instance, has already been approved in China by the end of June 2020 in China, followed by “Sputnik V” in August 2020 in Russia) that no one could expect in spring 2020.

Naturally, in 2020 governments found themselves in a position where they could only react to the dynamic development of the pandemic. I.e., lockdowns (with different intensities) and travel restrictions have been implemented regarding certain identification numbers (e.g., number of new infections, number of total infections, number of hospitalized people, number of people in intensive care units) and the dynamics without knowing any details concerning the *time horizon*. This is reflected by two problematic points in the relevant literature on COVID-19.

First, governments intend to take measures to control the pandemic in the best way. The term *best* in this context means that the performance of interventions is valued with respect to some objective function, which might include an economic (e.g., lost GDP per capita) as well as a health part (e.g., monetary valuation of lost lives). Due to the dynamic nature of a pandemic, the application of dynamic optimization or optimal control theory is suitable (see e.g., [3, 4]).

Second, the end of the pandemic in many other contributions (see below for a review of the relevant literature) is often identified with the availability of an effective vaccine. Neglecting regional differences (industrialized countries, emerging economies, and developing countries), the current situation illustrates that COVID-19 related problems, such as high infection numbers and congested intensive care (IC) units, are not resolved immediately with vaccination availability. Vaccination does not assure an instantaneous coverage of the population due to administration time and management issues that may arise. It may be a long process, and it is not necessarily organized at a constant rate. Lockdown still remains the most effective tool to oppose the virus, and it needs to be considered (probably with certain adaptations) at least for some time.

**Fig 1:**
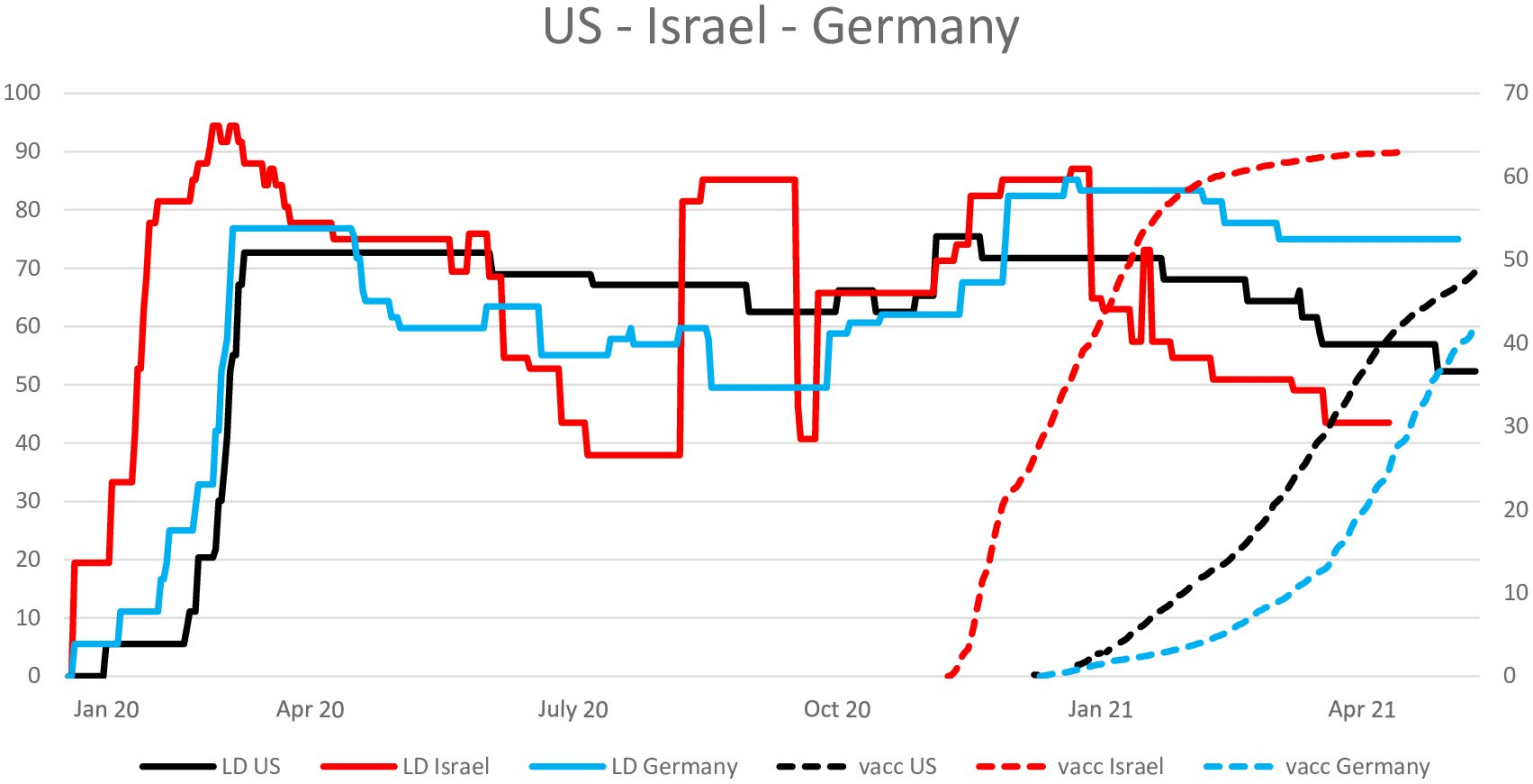
US, Israel and Germany. Lockdown intensity and proportion of vaccinated people.

The latter argument also becomes apparent in the data. Fig 1 plots the “intensity of the lockdown” (solid lines, values correspond to the left vertical axis) and the proportion of vaccinated people (dashed lines, values correspond to the right vertical axis) for US (black lines), Israel (red lines) and Germany (blue lines). The data has been taken from https://ourworldindata.org (last access May 15, 2021; see [5]). The intensity of the lockdown (referred to as “stringency index” in the data set) is measured from 0 to 100, where 0 means no lockdown at all and 100 lockdown of all nonessential parts of the economy. These three countries are highly developed and have the financial power to afford the necessary vaccine doses, but are heterogeneous concerning their vaccination strategies. While the US campaign has been quite quick and efficient, Germany faced a couple of problems at the beginning of the campaign much like numerous other European countries. Israel’s campaign has probably been the fastest in the world, resulting in an early relaxation of lockdown measures. Despite these differences, in all three countries, the lockdown was not relaxed immediately at the vaccine approval (which can also be observed for many other countries). Actually, in Israel and Germany it was even intensified. Relaxation in all three countries occurred at the time when a reasonable proportion of the population had been vaccinated (US and Germany: 15 − 20%, Israel: ≈40%) which, of course, goes along with a decrease of infection numbers and reduced pressure on IC units.

To overcome these crucial issues, we use a standard SIR model originated in the seminal paper [6], as already previously done in several mathematical economic papers on the COVID-19 pandemic (see the following paragraphs for a brief review), and include lockdown intensity as a policy measure (that has to be chosen optimally) as well as a stochastic arrival time of an efficient vaccination, that subdivides the time horizon into two stages.

In the stage before an efficient vaccination is available (from now on referred to as Stage 1) the lockdown is the only instrument of the government to push back the virus, and an additional research effort is employed towards the discovery of an effective vaccine. In the second stage, when a vaccine has been discovered (from now on referred to as Stage 2), the administration of the vaccine speeds up the immunization of the population. Lockdown measures can still be undertaken, but additional research effort is not necessary any more.

With this framework we aim to address the following *research questions*:

1. How does the optimal lockdown intensity change during Stage 1, when the vaccination approval is expected (compared to a situation without the expectation of a vaccine development)?
2. How should the optimal lockdown intensity be adapted from the vaccine approval time onwards?
3. How are the pandemic costs composed w.r.t. different approval times of a vaccination?

These questions are systematically addressed in the section “Numerical results” for different model scenarios. The thorough discussion gives important insights and extends the results of previous papers.

For the model setup, we adapt the baseline model proposed in [7]. From a mathematical point of view, any other optimal control model for the current pandemic (see the list of references) could be used. However, adding further compartments (i.e., state variables) or additional policy instruments (i.e., control variables) would neither change the qualitative behavior nor improve the intuitive understanding. Additional channels would cover up the effect of the stochastic arrival rate of a vaccination.

The literature on mathematical models of the COVID-19 pandemic is already quite rich. At this point, we focus the discussion on papers that are most related to our model. Most papers consider a macroeconomic objective function with lockdown as control variable but address different aspects of optimal policy interventions during the pandemic [7, 8] consider optimal lockdown policies over time. [9] extends the epidemiological model and considers a sophisticated SEIARD model. [10], on the other hand, analyzes the effect of quarantine. [11] includes the effect of social fatigue and endogenous treatment capabilities. [12–14] concentrate on the qualitative behavior of the solution and identify parameter regions where the optimal solution is qualitative stable. The parameter regions are separated by bifurcation curves where Skiba solutions or other interesting mathematical phenomena arise. [15] considers a transmission rate originating from a stochastic diffusion process, which can be influenced by confinement policies. [16] is the only paper that includes a stochastic time horizon within a SIR model with different age groups (deriving optimal interventions that may differ between the groups). However, in contrast to our contribution, the period after the vaccination arrival is not considered. [17] embeds work intensity, which is chosen on an individual basis, into a SIR model and derives the optimal lockdown policy for a given vaccination rate. The main finding is that the lockdown is relaxed when the vaccination effort is intensified (i.e., lockdown and vaccination are substitutes). This is contrasted by [18], where a systematic sensitivity analysis of the optimal lockdown intensity with respect to the vaccination rate and the valuation of a lost life is provided. In addition to the substitution property (as in [17]) also scenarios with lockdown and vaccination being complements are identified. Up to the best of our knowledge, [19] is the only paper that considers an (exogenous) stochastic arrival of a vaccine and the adaptation of the optimal lockdown policy afterward. However, the paper uses a different model setup and solution method, as well as a considerably different focus in their numerical examples.

Except [16, 19], the works mentioned above consider a fixed time horizon assuming that this immediate end of the pandemic is known a priori. As previously mentioned, this assumption is relaxed in our contribution within a model being truly simple in terms of the epidemiological dynamics. However, it allows to analyze the effect of a stochastic arrival rate of a vaccine and to work out the impact of different arrival times, which is not dealt with in [16, 19]. This is possible by adopting a novel approach for multi-stage optimal control models with random switching time as presented in [20]. Based on the deterministic reformulation of the objective function (see [21]), the problem is transformed into an age-structured optimal control problem. This approach allows treating both stages simultaneously (in contrast to the *traditional* method), thus implying a detailed characterization of the link between the two stages.

The rest of the paper is organized as follows. The following section introduces the model together with some analytic results. Numerical solutions for different cases concerning the speed of the vaccination campaign are presented in the section “Numerical results”. The last section concludes.

## The model

Within this section, we extend the well-known SIR model proposed in the seminal article [6] and improved in many other papers. We introduce the control variable *lockdown intensity* to fight the spread of the virus and a stochastic arrival time (i.e., a switch of the dynamics) of an efficient vaccine that separates the planning horizon into two stages. First, the adapted SIR dynamics are described; then the government’s objective function (i.e., costs) is defined. Finally, the full model is formulated.

### Dynamics

Consider an infinite planning period [0, + ∞) divided into two stages by the entrance of an effective vaccine at time *τ*. Assuming that such an entrance is a certain event (sooner or later), the arrival rate of *τ* is discussed below by the mean of a cumulative distribution function (see Eq (5)).

In Stage 1, i.e., in the interval [0, *τ*), no effective vaccine is available, therefore the two possible measures to fight the disease are the lockdown intensity, denoted by ℓ(*t*), and the additional research effort (research boost) towards the discovery of an effective vaccine, denoted by *r*(*t*). Both measures (entering non-linearly in the model) are continuous control variables and optimally set by the government. We assume that it is possible to close only nonessential parts of the economy. Essential parts (such as energy supply, health services, and basic food production) have to be kept open. This implies $\ell \left(t\right)\in \left[0,\bar{L}\right]$, where *ℓ*(*t*) = 0 corresponds to no lockdown and $\ell \left(t\right)=\bar{L}>0$ to full lockdown.

The total population *N*(*t*) is divided into the three common compartments, i.e., susceptibles *S*(*t*), infected *I*(*t*) and recovered *R*(*t*). In Stage 2 an additional compartment *V*(*t*) for vaccinated individuals is introduced. Due to the negligible effect on the pandemic, the non-COVID-19 mortality rate and the birth rate are ignored. As a result, the total population equals $N\left(t\right)=S\left(t\right)+I\left(t\right)+R\left(t\right)+I_{t\geq \tau }V\left(t\right)$, where $I_{t\geq \tau }$ denotes the usual indicator function (equal to 1 for *t* ≥ *τ*, zero otherwise).

At a lockdown intensity a proportion of *ℓ*, (1 − *θℓ*) is active and can transmit the virus, where *θ* ∈ [0, 1] is an exogenous measure of the lockdown effectiveness. Following [7] the transmission rate is defined as *β*(*ℓ*) = *β*_0_(1 − *θℓ*)^2^, where *β*_0_ > 0 denotes the transmission rate without lockdown. Thus, during Stage 1 (i.e., *t* ∈ [0, *τ*)) the number of susceptibles evolves according to

$$\begin{array}{c} \dot{S}\left(t\right)=-\beta \left(\ell \left(t\right)\right)\frac{S(t)I(t)}{N(t)},\;S\left(0\right)=S_{0},\;\text{for}\;t<\tau , \end{array}$$ (1)

where *S*_0_ is the initial value of the compartment. The increase of the infected due to new infections equals the decrease of the susceptibles. On the other hand, the infected decrease because of COVID-19 related deaths and recoveries. Let $\frac{1}{\gamma }$ denote the average dwell time of individuals in *I*(*t*), then *γ* equals the percentage of infected leaving the compartment at any point in time, i.e., $\gamma =\frac{\left(\text{recovered}+\text{dead}\right)}{\text{infected}}$. For the COVID-19 related death rate, we follow [7] and assume the following linear increasing form

$$\begin{array}{c} \varphi \left(I\left(t\right)\right)=\gamma \cdot \left(\bar{\varphi }+\kappa I\left(t\right)\right), \end{array}$$ (2)

where the assumption $(\bar{\phi }+\kappa I\left(t\right))<1$ guarantees a positive recovery rate equal to $\gamma (1-(\bar{\phi }+\kappa I\left(t\right)))$. The death rate is increasing in *I* due to the congestion effects of the health sector (for dramatic examples we refer to Lombardia in Italy or New York City both in spring 2020, or Brazil in spring 2021). Several contributions use a similar form, see e.g., [12–14] who assume an excess death rate if the ICU capacity is exceeded.

Therefore, the infected dynamics during both stages is

$$\begin{array}{c} \dot{I}\left(t\right)=\beta \left(\ell \left(t\right)\right)\frac{S(t)I(t)}{N(t)}-\gamma I\left(t\right),\;I\left(0\right)=I_{0}, \end{array}$$ (3)

where *I*_0_ contains the initial number of infected individuals.

The size of the total population at any *t* diminishes by COVID-19 deaths. This implies the following dynamics for both stages

$$\begin{array}{c} \dot{N}\left(t\right)=-\varphi \left(I\left(t\right)\right)I\left(t\right),\;N\left(0\right)=N_{0}, \end{array}$$ (4)

with an initial population size of *N*_0_. The number of recovered people *R*(*t*) in both stages can be directly obtained by $R\left(t\right)=N\left(t\right)-S\left(t\right)-I\left(t\right)-I_{t\geq \tau }V\left(t\right)$.

The time instant *τ*, when an efficient vaccination becomes *available*, is assumed to be an absolute continuous random variable. Let us define by *Z*(*t*) the probability of discovering an effective vaccine after *t*. In other words, the probability of remaining in Stage 1 until *t*. We formalize this probability throughout the complementary cumulative distribution function (tail distribution)

$$\begin{array}{c} Z(t)=Prob\{\tau >t\}. \end{array}$$ (5)

As a result, the corresponding switching rate is obtained by

$$\begin{array}{c} \frac{-\dot{Z}\left(t\right)}{Z(t)}=\eta \left(r\left(t\right),t\right). \end{array}$$ (6)

Since *Z*(0) = *Prob*{*τ* > 0} = 1 holds naturally, this distribution function satisfies the following Cauchy problem

$$\begin{array}{c} \dot{Z}\left(t\right)=-\eta \left(r\left(t\right),t\right)Z\left(t\right),\;Z\left(0\right)=1. \end{array}$$ (7)

We assume *η*(⋅, *t*) to be positive, continuous, and to depend on the additional research effort *r*(*t*), which is a control variable in Stage 1. Note that *r*(*t*) ∈ [0, 1], i.e., *r*(*t*) = 0 and *r*(*t*) = 1 correspond to the cases without and with maximal available additional research effort respectively. Note that research effort cannot exceed a certain level at least on the medium term due to constraints on the availability of experienced researchers, research facilities, etc. Consequently, *η*(0, *t*) captures the switching rate resulting from base research effort (spent in R&D on a regular basis, without any additional research effort, i.e., with *r* = 0).

We assume that the research boost *r*(*t*), aimed at finding an effective vaccine, accelerates its development with efficacy *η*_1_ > 0. Thus, the switching rate depends on *r*(*t*) as follows,

$$\begin{array}{c} \eta \left(r\left(t\right),t\right)=p\left(t\right)(\eta_{0}+\eta_{1}r\left(t\right)), \end{array}$$ (8)

with *η*_0_ > 0, and where *p*(*t*) is an increasing time dependent function such that *p*(0) ∈ (0, 1) and lim_*t*→∞_ *p*(*t*) = 1. For the numerical calculations in Section “Numerical results”, we are using a Gompertz sigmoid function, i.e., $p\left(t\right)=e^{-p_{1}e^{-p_{2}t}}$ with *p*_1_, *p*_2_ > 0 (see Table 2 for the parameter values). This functional specification guarantees not only a switching rate that increases in *r*(*t*), but also a time-dependent learning effect (non-autonomous *p*(*t*)) resulting in a more efficient use of research efforts.

At the time instant *τ* a vaccine is developed and becomes available, thus marking the start of the vaccination of the population. We assume that the cost for production and administration of the vaccine (research costs are already covered in Stage 1) are minuscule compared to costs resulting from lockdown measures and lost lives. This implies that at every *t* as many people as possible (with respect to production and administration capacities) get vaccinated. Hence the number of vaccinations per unit of time $\alpha (\hat{t})$ (where $\hat{t}$ corresponds to the time after the switch, i.e., $\hat{t}≔t-\tau$) follows the exogenously given availability of vaccination after discovery. We assume that *α*(⋅) is an increasing and concave function over time, i.e., $\alpha^{\prime }\left(\hat{t}\right)>0$ and $\alpha^{\prime \prime }\left(\hat{t}\right)<0$. For the functional specification, we propose the following form

$$\begin{array}{c} \alpha \left(\hat{t}\right)=\frac{\alpha_{1}\hat{t}+\alpha_{2}}{\hat{t}+\alpha_{3}}, \end{array}$$ (9)

with positive parameters *α*_*i*_ > 0 (*i* = 1, 2, 3); *α*_1_*α*_3_ > *α*_2_ guarantees that *α*(*t*) is increasing and concave. At the switch $\alpha \left(\tau -\tau \right)=\alpha \left(0\right)=\frac{\alpha_{2}}{\alpha_{3}}$ vaccination doses are available. This number increases up to the maximum level of *α*_1_, which is the limit of the above expression.

Although people are vaccinated as quickly as possible starting at *τ*, as discussed in the introduction (and as observed in lots of countries in the first half of 2021), the lockdown is still an indispensable tool to control the pandemic since people cannot be vaccinated fast enough. We assume that susceptible and recovered people are vaccinated without any prioritization. After getting the vaccine, people enter the compartment of vaccinated people *V*(*t*), which is the absorbing state in our model. As a result, the dynamics of *S*(*t*) and *R*(*t*) in Stage 2 read

$$\begin{array}{ccc} \dot{S}\left(t\right) & = & -\beta \left(\ell \left(t\right)\right)\frac{S(t)I(t)}{N(t)}-\alpha \left(t-\tau \right)\frac{S}{S+R}I_{S+R>0}\\ \dot{I}\left(t\right) & = & \beta \left(\ell \left(t\right)\right)\frac{S(t)I(t)}{N(t)}-\gamma I\left(t\right). \end{array}$$ (10)

where the indicator function guarantees that vaccination ends if everybody is vaccinated. *V*(*t*) just collects all vaccinated people in Stage 2

$$\begin{array}{c} \dot{V}\left(t\right)=\alpha \left(t-\tau \right)I_{S+R>0},\;V\left(\tau \right)=0. \end{array}$$ (11)

### Costs—objective function

The decision maker in our model is the government balancing (i) the costs of lost lives and (ii) the costs of lockdown and subsidies in the R&D sector (aiming at accelerating the vaccine development). Therefore, the objective function consists of an economic (lockdown, research subsidies) and a health economic part (lost lives), both measured in GDP (gross domestic product) per day. All objectives in Stage 1 are represented by the function

$$\begin{array}{c} g_{1}\left(I\left(t\right),\ell \left(t\right),r\left(t\right)\right)≔c_{h}\left(I\left(t\right)\right)+c_{\ell }\left(\ell \left(t\right)\right)+c_{r}\left(r\left(t\right)\right). \end{array}$$ (12)

Here *c*_*h*_(*I*(*t*)) ≔ *ψ* ⋅ *φ*(*I*(*t*)) ⋅ *I*(*t*) denotes health economic costs, i.e., the costs of lost lives weighted with the value of a statistical life *ψ*. The range of *ψ* in the literature ranges from 20 in [7] to 150 in [22]. In [12, 13] this value is used as a bifurcation parameter in a sensitivity analysis in a different model setting with a different research focus.

Lockdown costs are assumed to be quadratic, i.e.,

$$\begin{array}{c} c_{\ell }\left(\ell \left(t\right)\right)≔w\ell^{2}\left(t\right) \end{array}$$ (13)

with a positive parameter *w*. This results from higher marginal costs as lockdown becomes more intense, due to the interconnection of the economy. Note that the definition of lockdown costs varies in the corresponding literature. While e.g., [7] assumes linear costs, e.g., [12, 13] assume non-linear costs that also depend on the available workforce (i.e., *N*(*t*) diminished by *I*(*t*)). Moreover, we are relaxing this assumption in the section “Robustness check” and allow for convex-concave costs.

Similar to the lockdown costs, the research costs are assumed to be quadratic *c*_*r*_(*r*(*t*)) = *c*_0_*r*^2^(*t*), with a positive parameter *c*_0_. This appropriately reflects reality since the research effort enters linearly in the switching rate and research projects are funded in order with their priority and probability of success.

The costs in Stage 2 are analogous to the ones in Stage 1, they only differ for the lack of additional research efforts (which are assumed to be zero after the arrival of an efficient vaccine), i.e.,

$$\begin{array}{c} g_{2}\left(I\left(t\right),\ell \left(t\right)\right)≔c_{h}\left(I\left(t\right)\right)+c_{\ell }\left(\ell \left(t\right)\right). \end{array}$$ (14)

Note that we do not consider vaccination costs explicitly, while they could be argued to be included. However, as discussed before, vaccinations are given at the maximum pace at any *t* (due to the fact that the corresponding costs are negligible) implying that the optimal results would not change.

As the decision maker aims for the minimization of the expected aggregated discounted costs over an infinite time horizon (taking the stochasticity of the switching time *τ* into account), the objective function can be written as

$$\begin{array}{c} J^{*}\left(X\left(0\right)\right)≔\underset{\ell (t),r(t)}{\text{min}}\;E_{\tau }[\int_{0}^{\tau }\;e^{-\rho t}g_{1}\left(I\left(t\right),\ell \left(t\right),r\left(t\right)\right)\;\;dt+\int_{\tau }^{+\infty }\;e^{-\rho t}g_{2}\left(I\left(t\right),\ell \left(t\right)\right)\;\;dt]. \end{array}$$ (15)

Thereby *ρ* is the discount rate and *X*(*t*) is a vector of the compartments *S*(*t*), *I*(*t*), and *N*(*t*).

Following [20], the objective function (15) can be reformulated as

$$\begin{array}{c} J^{*}\left(X\left(0\right)\right)=\underset{\ell (t),r(t)}{\text{min}}\int_{0}^{\infty }\;e^{-\rho t}Z\left(t\right)[g_{1}\left(I\left(t\right),\ell \left(t\right),r\left(t\right)\right)+\eta \left(r\left(t\right),t\right)J_{2}^{*}\left(X\left(t\right),t\right)]\;\;dt \end{array}$$ (16)

where $J_{2}^{*}\left(X\left(t\right),t\right)$ denotes the optimal value (in mathematical terms referred to as value function, see [4]) of Stage 2 given a vaccine approval at *t* (with state variables *X*(*t*) at *t*).

### Full model

Using the subscripts 1 and 2 to refer to the (state and control) variables corresponding to Stage 1 or 2 respectively, the problem in Stage 1 can be written as

$$\begin{array}{ccc} J^{*}\left(X\left(0\right)\right) & = & \underset{\ell_{1}\left(t\right),r_{1}\left(t\right)}{\text{min}}\int_{0}^{\infty }e^{-\rho t}Z_{1}\left(t\right)[g_{1}\left(I_{1}\left(t\right),\ell_{1}\left(t\right),r_{1}\left(t\right)\right)+\eta \left(r_{1}\left(t\right),t\right)J_{2}^{*}\left(X_{1}\left(t\right),t\right)]\;\;dt\\ s.t.{\dot{S}}_{1}\left(t\right) & = & -\beta \left(\ell_{1}\left(t\right)\right)\frac{S_{1}\left(t\right)I_{1}\left(t\right)}{N_{1}\left(t\right)}\\ {\dot{I}}_{1}\left(t\right) & = & \beta \left(\ell_{1}\left(t\right)\right)\frac{S_{1}\left(t\right)I_{1}\left(t\right)}{N_{1}\left(t\right)}-\gamma I_{1}\left(t\right)\\ {\dot{N}}_{1}\left(t\right) & = & -\varphi \left(I_{1}\left(t\right)\right)I_{1}\left(t\right)\\ {\dot{Z}}_{1}\left(t\right) & = & -\eta \left(r_{1}\left(t\right),t\right)Z_{1}\left(t\right) \end{array}$$ (17)

with initial conditions

$$\begin{array}{c} S_{1}\left(0\right)=S_{0},\;I_{1}\left(0\right)=I_{0},\;N_{1}\left(0\right)=N_{0},\;Z_{1}\left(0\right)=1. \end{array}$$ (18)

The problem in Stage 2 can be written analogously, and defines $J_{2}^{*}\left(X\left(t\right),t\right)$ which occurs in the objective function of Stage 1. Since for every possible realization of *τ* the value function is derived from an optimal control problem, *x*_2_(*t*, *τ*) indicates a (state and control) variable of Stage 2 at *t*, given a vaccine approval at *τ* (*τ* ≤ *t*). This yields

$$\begin{array}{ccc} J_{2}^{*}\left(X\left(\tau \right),\tau \right) & = & \underset{\ell_{2}\left(t,\tau \right)}{\text{min}}\int_{\tau }^{\infty }e^{-\rho t}g_{2}\left(I_{2}\left(t,\tau \right),\ell_{2}\left(t,\tau \right)\right)\;\;dt\\ \text{s}.\text{t}.\;{\dot{S}}_{2}\left(t,\tau \right) & = & -\beta \left(\ell_{2}\left(t,\tau \right)\right)\frac{S_{2}\left(t,\tau \right)I_{2}\left(t,\tau \right)}{N_{2}\left(t,\tau \right)}-\alpha \left(t-\tau \right)\frac{S_{2}\left(t,\tau \right)}{S_{2}\left(t,\tau \right)+R_{2}\left(t,\tau \right)}I_{S_{2}\left(t,\tau \right)+R_{2}\left(t,\tau \right)>0}\\ {\dot{I}}_{2}\left(t,\tau \right) & = & \beta \left(\ell_{2}\left(t,\tau \right)\right)\frac{S_{2}\left(t,\tau \right)I_{2}\left(t,\tau \right)}{N_{2}\left(t,\tau \right)}-\gamma I_{2}\left(t,\tau \right)\\ {\dot{N}}_{2}\left(t,\tau \right) & = & -\varphi \left(I_{2}\left(t,\tau \right)\right)I_{2}\left(t,\tau \right)\\ {\dot{V}}_{2}\left(t,\tau \right) & = & \alpha \left(t-\tau \right)I_{S_{2}\left(t,\tau \right)+R_{2}\left(t,\tau \right)>0} \end{array}$$ (19)

with initial conditions

$$\begin{array}{c} S_{2}\left(\tau ,\tau \right)=S_{1}\left(\tau \right),\;I_{2}\left(\tau ,\tau \right)=I_{1}\left(\tau \right),\;N_{2}\left(\tau ,\tau \right)=N_{1}\left(\tau \right),\;V_{2}\left(\tau ,\tau \right)=0. \end{array}$$ (20)

The dot-notation in Stage 2 refers to the derivative with respect to time, i.e., $\dot{x}\left(t,\tau \right)=\frac{dx(t,\tau )}{dt}$, while *τ* remains constant.

Clearly, the number of recovered people in both stages can be derived by the identity *N* = *S* + *I* + *R*(+*V*). Note that in the problem of Stage 2 the vaccine arrival time *τ* is only a parameter (therefore entering as parameter in the value function $J_{2}^{*}\left(X\left(\tau \right),\tau \right)$), whereas in the objective function of Stage 1 the value function of Stage 2, $J_{2}^{*}\left(X_{1}\left(t\right),t\right),$ is evaluated for every *t*, as a possible arrival time. Table 1 summarizes all variables and functions of the model for both stages, at a glance.

**Table 1:** Overview of Notation (*i* = 1, 2).

Notation	Description	Stage 1	Stage 2
**Controls**:			
*ℓ*_*i*_	lockdown intensity	X	X
*r*_1_	research effort (development of vaccination)	X	-
**States**:			
*S*_*i*_	susceptibles	X	X
*I*_*i*_	infected	X	X
*R*_*i*_	recovered	X	X
*V*_2_	vaccinated	-	X
*N*_*i*_	total population	X	X
*Z*_1_	probability of vaccine approval after *t*	X	-
**Functions**:			
*η*(*r*_1_, *t*)	switching rate	X	-
*α*(*t* − *τ*)	number of vaccinations per unit of time	-	X
*β*(*ℓ*_*i*_)	transmission (infection) rate	X	X
*φ*(*I*_*i*_)	fatality rate	X	X
*γ* − *φ*(*I*_*i*_)	recovery rate	X	X

### Solution and economic intuition

For the derivation of the first-order conditions, we first transform the model into an age-structured optimal control model (see [20]). Then the age-structured Maximum Principle (see [23]) is applied. This representation offers some advantages compared to the standard approach presented in [21], as discussed in [20].

The transformation of Eqs (17)–(19) into the age-structured form, as well as the derivation of the optimality conditions, is quite technical and deferred to the S1 Appendix.

The optimal values of the control variables are summarized in the following Theorem.

**Theorem 1** *Consider the above multi-stage optimal control problem with random switching time* (17)–(19). *Then, assuming the existence of an optimal solution, the optimal research efforts in Stage 1 and the optimal lockdown levels in both stages are given by*

$$\begin{array}{ccc} r_{1}^{*}\left(t\right) & = & \text{min}\{\text{max}\{\frac{\eta_{1}p\left(t\right)(\xi_{Z}\left(t,t\right)-\lambda_{Z}\left(t\right))}{2c_{0}},0\},1\},\\ \ell_{1}^{*}\left(t\right) & = & \text{min}\{\text{max}\{\frac{1}{\theta }\frac{(\lambda_{I}\left(t\right)-\lambda_{S}\left(t\right))\beta_{0}\theta \frac{S_{1}\left(t\right)I_{1}\left(t\right)}{N_{1}\left(t\right)}}{(\lambda_{I}\left(t\right)-\lambda_{S}\left(t\right))\beta_{0}\theta \frac{S_{1}\left(t\right)I_{1}\left(t\right)}{N_{1}\left(t\right)}-Z_{1}\left(t\right)\frac{w}{\theta }},0\},\bar{L}\},\\ \ell_{2}^{*}\left(t,\tau \right) & = & \text{min}\{\text{max}\{\frac{1}{\theta }\frac{(\xi_{I}\left(t,\tau \right)-\xi_{S}\left(t,\tau \right))\beta_{0}\theta \frac{S_{2}\left(t,\tau \right)I_{2}\left(t,\tau \right)}{N_{2}\left(t,\tau \right)}}{(\xi_{I}\left(t,\tau \right)-\xi_{S}\left(t,\tau \right))\beta_{0}\theta \frac{S_{2}\left(t,\tau \right)I_{2}\left(t,\tau \right)}{N_{2}\left(t,\tau \right)}-Z_{1}\left(t\right)\eta \left(t\right)\frac{w}{\theta }},0\},\bar{L}\}, \end{array}$$ (21)

*where* λ_*y*_ (*ξ*_*y*_) *denotes the adjoint variable of Stage 1 (Stage 2) of a given state variable y (for the corresponding adjoint equations we again refer to the* S1 Appendix*)*.

Giving a more detailed discussion on λ_*Z*_(*t*) and *ξ*_*Z*_(*t*, *t*): λ_*Z*_(*t*) denotes the adjoint variable of *Z*_1_(*t*), which is the probability that the vaccine is approved after *t*. *ξ*_*Z*_(*t*, *t*) denotes the adjoint variable of *Z*_2_(*t*, *t*), which is the probability density that the vaccine is approved at *t*.

Proof:

Manipulating the general first order optimality conditions (see S1 Appendix), directly leads to the presented expression for the optimal controls. The max and min operators inside the expressions guarantee that the control variables stay within their admissible regions.

The formal expression of Theorem 1 can be interpreted as follows:

- Optimal research effort (Stage 1): In case the fraction inside the brackets is positive and below one (i.e., $r_{1}^{*}\left(t\right)\in \left(0,1\right)$), the max and min operators can be omitted. Then the optimal value equals the marginal effect of research subsidies over their marginal costs. The marginal effect comprises of the product of the marginal switching rate (w.r.t. research effort) and the shadow price of a switch from Stage 1 to Stage 2 at *t* (i.e., the difference of the corresponding shadow prices *ξ*_*Z*_(*t*, *t*) and λ_*Z*_(*t*)).
  Therefore $r_{1}^{*}\left(t\right)$ increases if the marginal effect of the switching rate increases, if the marginal value of the vaccine approval increases, or if the marginal cost decreases.
- Optimal lockdown intensity without vaccination (Stage 1): The second-order optimality conditions (see S1 Appendix) imply that the denominator of the fraction inside the brackets is always negative. In the case of an inner solution (i.e., $\ell_{1}^{*}\left(t\right)\in \left(0,\bar{L}\right)$) the term λ_*I*_ − λ_*S*_, which can be interpreted as the shadow price of one individual getting infected, is strictly negative. Thus the optimal value equals the marginal effect on the epidemiological dynamics (numerator) over the total effect (denominator) divided by the marginal effectiveness of the lockdown (*θ*). The marginal effect on the dynamics (numerator) comprises of the shadow price of a person getting infected (difference of the shadow prices of the corresponding compartments) weighted by the corresponding probability and the marginal effect of the lockdown intensity. To get the total effect (denominator), the marginal relative cost of lockdown efficiency (weighted by the probability that the vaccine has not been approved at *t*) is added to the effect on the epidemiological dynamics. This fraction is related to the effectiveness of the lockdown (i.e., division by *θ*). As a result, the lockdown increases in the effect on the dynamics (either by the shadow price of a person getting infected or by the corresponding probability) and decreases in the costs.
- Optimal lockdown intensity with vaccination (Stage 2): The interpretation of $\ell_{2}^{*}\left(t,\tau \right)$ is analogous to that of $\ell_{1}^{*}\left(t\right)$.

In addition to this interpretation Theorem 1 can be used for analyzing the lockdown intensity at the vaccination approval, which is not a-priori clear. Intuitively, one would expect that the lockdown will be relaxed or at most remain at the same level. However, as already discussed in the introduction, in the beginning of 2021 most countries in Europe reacted differently. These decisions can potentially be supported by our framework. In particular, the following theorem formalizes conditions for the adaptation of the lockdown intensity at the vaccine approval.

**Theorem 2** *Consider the multi-stage optimal control problem with random switching time* Eqs (17)–(19) *and the optimal lockdown intensities given in Theorem 1. At the (stochastic) time of a vaccine approval τ the lockdown intensity is in general non-continuous (disruptive) and*

$$\begin{array}{c} \begin{array}{ccc} (disruptively)\;increasing & \Leftrightarrow & \frac{\xi_{I}\left(\tau ,\tau \right)-\xi_{S}\left(\tau ,\tau \right)}{\lambda_{I}\left(\tau \right)-\lambda_{S}\left(\tau \right)}>\eta \left(r_{1}\left(\tau \right),\tau \right)\\ continuous & \Leftrightarrow & \frac{\xi_{I}\left(\tau ,\tau \right)-\xi_{S}\left(\tau ,\tau \right)}{\lambda_{I}\left(\tau \right)-\lambda_{S}\left(\tau \right)}=\eta \left(r_{1}\left(\tau \right),\tau \right)\\ (disruptively)\;decreasing & \Leftrightarrow & \frac{\xi_{I}\left(\tau ,\tau \right)-\xi_{S}\left(\tau ,\tau \right)}{\lambda_{I}\left(\tau \right)-\lambda_{S}\left(\tau \right)}<\eta \left(r_{1}\left(\tau \right),\tau \right) \end{array} \end{array}$$ (22)

*given that the total effect of the lockdown intensity of Stage 1 and Stage 2 at τ has the same sign (a different sign just reverses the inequalities)*.

Proof:

From Theorem 1 we obtain

$$\begin{array}{ccc} \ell_{2}^{*}\left(\tau ,\tau \right)-\ell_{1}^{*}\left(\tau \right) & = & \beta_{0}\frac{S_{1}\left(\tau \right)I_{1}\left(\tau \right)}{N_{1}\left(\tau \right)}(\frac{\xi_{I}\left(\tau ,\tau \right)-\xi_{S}\left(\tau ,\tau \right)}{(\xi_{I}\left(\tau ,\tau \right)-\xi_{S}\left(\tau ,\tau \right))\beta_{0}\theta \frac{S_{1}\left(\tau \right)I_{1}\left(\tau \right)}{N_{1}\left(\tau \right)}-Z_{1}\left(\tau \right)\eta \left(\tau \right)\frac{w}{\theta }}\\ & & \;\;\;-\frac{\lambda_{I}\left(\tau \right)-\lambda_{S}\left(\tau \right)}{(\lambda_{I}\left(\tau \right)-\lambda_{S}\left(\tau \right))\beta_{0}\theta \frac{S_{1}\left(\tau \right)I_{1}\left(\tau \right)}{N_{1}\left(\tau \right)}-Z_{1}\left(\tau \right)\frac{w}{\theta }}), \end{array}$$ (23)

for $\ell_{1}^{*}\left(\tau \right),\ell_{2}^{*}\left(\tau ,\tau \right)\in \left(0,\bar{L}\right)$ and accordingly for solutions on the boundary. Manipulation of this expression proves the assertion of the theorem.

According to Theorem 2 the adaptation of the lockdown intensity at the time of the vaccine approval depends on the relation of the shadow prices of a new infected person in Stage 2 and Stage 1 at *τ*. If the ratio exceeds the approval rate *η*(⋅) at *τ*, new infections have a stronger effect with the availability of a vaccine than they would have without. Consequently, the lockdown intensity has to be intensified disruptively at *τ*, which seems to be counter intuitive at first glance. However, the reason is hidden in the definition of the shadow price of new infections, which includes the net value of costs (i.e., lockdown, lost lives) and the effect on the dynamics of the epidemic. Therefore, in Stage 1 it might be more costly to keep a lot of people in the *S* compartment since everyone has to pass the *S* − *I* − *R*-route (while keeping *φ*(*I*(*t*)) on an acceptable level) to achieve immunity. On the other hand, if the vaccine is available, susceptibles can pass directly from the *S* to the *V* compartment by getting vaccinated. Thus, with a more intensive lockdown it pays off to keep more people in the *S* state (i.e., keeping them healthy), pass them directly to *V* and therefore save lives (lowering costs). In other words, a vaccination works as a kind of shortcut in the SIR dynamics. A more intense lockdown enables more people to make use of it.

The interpretation of a continuous and disruptive decrease of the lockdown intensity (second and third line of Eq (22)) is analogous.

## Numerical results

In this section, we present some numerical results obtained by applying the Pontryagin Maximum Principle (PMP) (see S1 Appendix for the analytical expressions). Most of the parameters’ values for our baseline calibration are taken from [7] and other models of the related literature. We report the complete list of parameters in Table 2, with time being measured in years. Note within the plots that the time axis is scaled in months for enhanced readability.

**Table 2:** Summary of parameter values (per year).

Parameter	Value	Description
*S*_0_	0.99	initial proportion of susceptibles in population
*I*_0_	0.01	initial proportion of infected in population
*N*_0_	1	initial population
$\bar{L}$	0.7	maximal lockdown intensity
*θ*	0.5	lockdown efficacy
*β*_0_	0.13 * 365	transmission rate without a lockdown
*γ*	$\frac{1}{18}*365$	recovery rate (including COVID-19 deaths)
$\bar{\varphi }$	0.0068	COVID-19 fatality rate
*κ*	0.034	coefficient for additional fatality rate (congestion of health care system)
*α*_1_, *α*_2_, *α*_3_	4, 0.1, 1	parameters concerning the availability of the vaccines
*ψ*	40	value of a statistical life (in terms of GDP)
*w*	1	lockdown cost parameters (in terms of GDP)
*η*_0_	$\frac{1}{1.5}$	parameter of the switching rate
*η*_1_	4 − *η*_0_	effectiveness of additional research effort
*p*1, *p*2	*e*, 3	parameters concerning the switching rate (Gompertz sigmoid)
*c*_0_	0.013	research effort cost parameters (in terms of GDP)
*ρ*	0.05	discount rate

Unlike [7], where the proportion *S*_0_ of susceptibles in the population is set equal to 0.97, we fix it equal to 0.99, so that *S*_0_ and *I*_0_ sum up to 1 (and no initial recovered are considered, *R*_0_ = 0) as in [12].

The parameters concerning research and vaccination must be explained in more detail. Up to the best of our knowledge the effect of research on the discovery of a vaccine has not been considered by other papers. As already explained after the definition of the switching rate Eq (8), we use a Gompertz sigmoid function for *p*(*t*) with *p*_1_ = *e* and *p*_2_ = 3, such that we end up with

$$\begin{array}{c} p\left(t\right)=e^{-e^{1-3t}}. \end{array}$$ (24)

This essentially means that research efforts are rather ineffective at the start of the pandemic (i.e., at *t* = 0), and develop their effectiveness soon (e.g., *p*(0.5) = 54.5% after half a year, *p*(1) = 87.3% after one year). For the base research efficiency *η*_0_ and the efficacy of the research boost *η*_1_ no data is available. We have chosen $\eta_{0}=\frac{1}{1.5}$ and *η*_1_ = 4 − *η*_0_. This reflects a situation in which the probability of a vaccine being approved within the first year without an additional research boost (i.e., *r*_1_(*t*) = 0 for all *t*) is only about 30%. On the other hand, for a maximal possible research boost (i.e., *r*(*t*) = 1 for all *t*) the probability for the approval within one year increases to more than 80%. We are aware that these parameters can hardly be validated. However, several numerical runs with different values implied that the results are qualitatively very robust with respect to these parameters.

**Fig 2:**
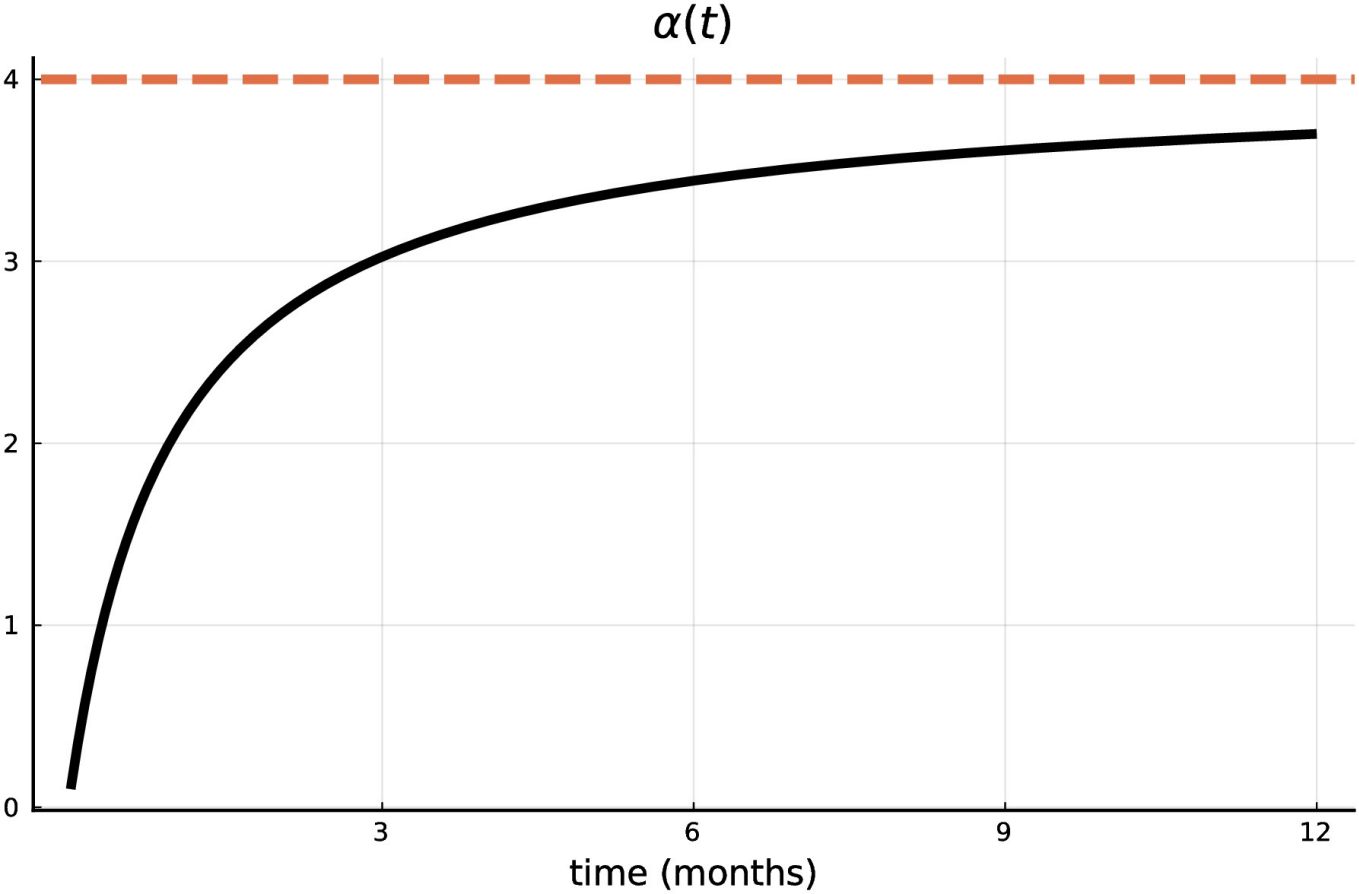
Number of vaccinations per unit of time.

Fig 2 plots the benchmark availability of the number of vaccinations per unit of time (modeled according to Eq (9)), where we assume that the whole population can be vaccinated in a quarter of a year (i.e., *α*_1_ = 4) at the maximum intensity, i.e., if *t* tends to +∞. Initially the campaign starts with 10% intensity (*α*_2_ = 0.1 and *α*_3_ = 1). This means that the whole population can in fact be vaccinated within about 10 months. This specification (used as benchmark scenario in the first subsection) reflects a typical situation in Europe, however, these numbers can vary across countries. Hence, a rather hypothetical example is studied in the second subsection, in which *α*(*t*) is increased proportionally such that the whole population can be vaccinated within one month.

For the numerical solution, the age-structured formulation of problem in Eqs (17)–(19) was used (see S1 Appendix). As already mentioned, this includes the advantage to solve both stages simultaneously, as one single problem instead of two sequential ones, i.e., first deriving Stage 2 for all possible values of the state variables at any *τ*; followed by Stage 1. An age-structured optimal control model implies the solution of a system of partial differential equations, which makes it difficult to use a standard boundary value solver. Thus, we use the established gradient optimization algorithm presented in [24]. After choosing initial guesses for the control variables, the algorithm uses the gradient to update the control variables iteratively until they converge to an optimum.

**Fig 3:**
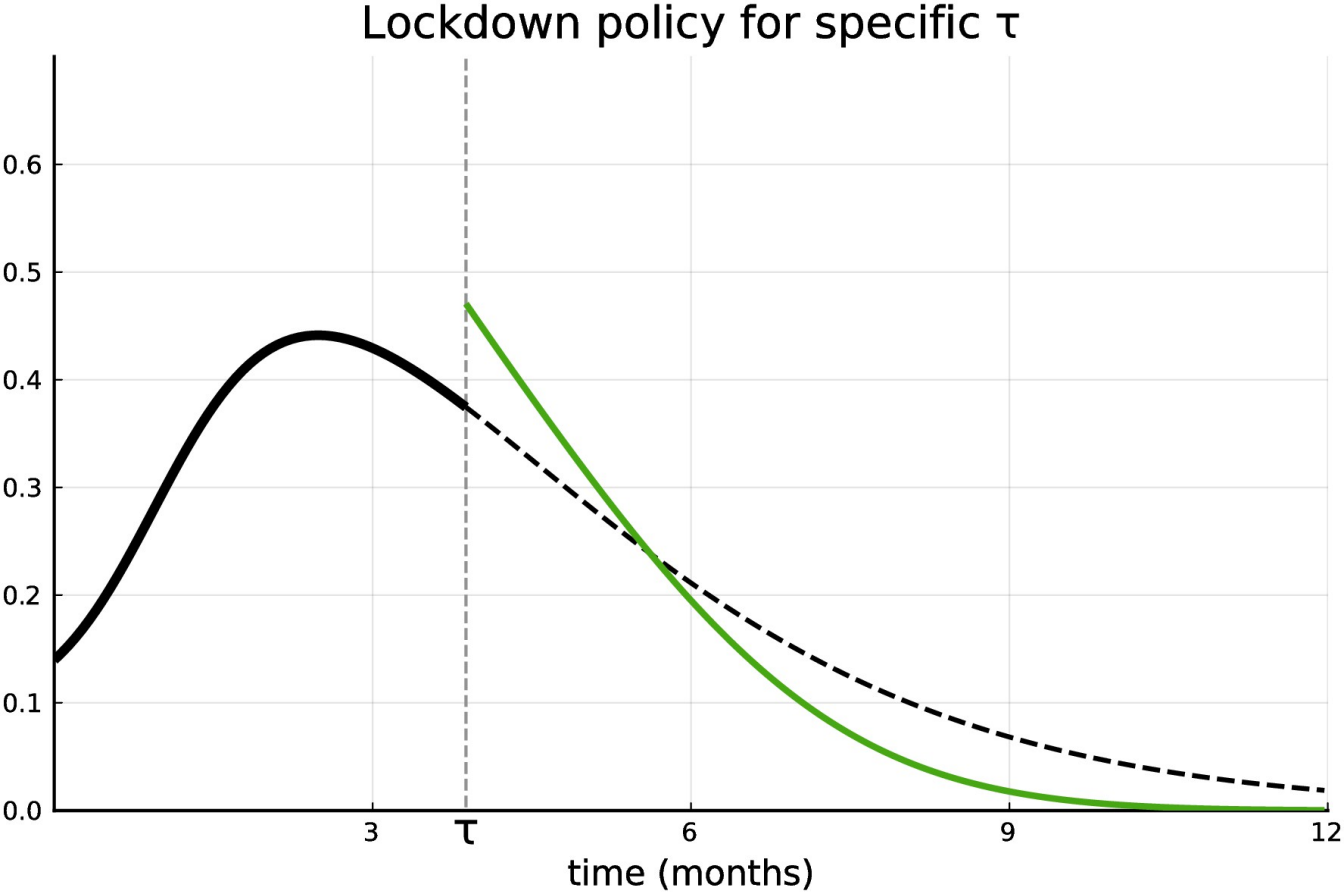
Lockdown intensity for approval at *τ* = 4. Lockdown intensity without vaccination (black line, Stage 1) and with vaccination (green line).

Before we start to discuss the specific results, we observe that with the parameters’ values declared above, the SOCs and the optimality conditions (see S1 Appendix) are fulfilled. Fig 3 introduces the way the results are presented in the following subsections. The horizontal and vertical axes denote time (in months) and lockdown intensity respectively. The black line (subdivided into a solid and a dashed part) shows the optimal lockdown intensity in case the vaccination was not approved until *t* (i.e., lockdown intensity of Stage 1). A vaccination may be approved at any time. In the current figure the optimal lockdown intensity of Stage 2 is only plotted for the case that the approval happens after approximately $\bar{\tau }\approx 4$ months (green line). Therefore, the optimal lockdown intensity given the approval at $\bar{\tau }$ (which is a specific realization of the random variable *τ*) follows the solid black line until $\bar{\tau }$ (i.e., *ℓ*_1_(*t*) for $t\in [0,\bar{\tau })$) and the green line afterwards (i.e., $\ell_{2}\left(t,\bar{\tau }\right)$ for $t\in [\bar{\tau },\infty )$). Thus at the approval of the vaccine at $\bar{\tau }$ the lockdown intensity jumps upwards. Before and after $\bar{\tau }$ the lockdown intensity changes continuously (as implied by the Maximum Principle). In the figures presenting results in the following subsections the black line always represents trajectories of Stage 1, whereas the coloured ones correspond to the trajectories of Stage 2 for different realizations of *τ*. In all figures of the following subsections, when presenting results, the black line will represent trajectories of Stage 1, whereas the coloured lines will correspond to the trajectories of Stage 2 for different realizations of *τ*.

In the first subsection, the benchmark scenario is presented with two different assumptions concerning the speed of the vaccination campaign. In Scenario 1 we assume the population to be vaccinated within 10 months, which seems to be pretty realistic considering many European countries. Meanwhile in Scenario 2 the population will be vaccinated within about one month.

In the second subsection, we compare the anticipative behavior of our model (i.e., lockdown measure during Stage 1) with the behavior of a myopic government, which does not include the arrival of a vaccination in the decision process.

### Optimal lockdown intensity before and after vaccine approval

#### Scenario 1: Vaccination within 10 months

**Fig 4:**
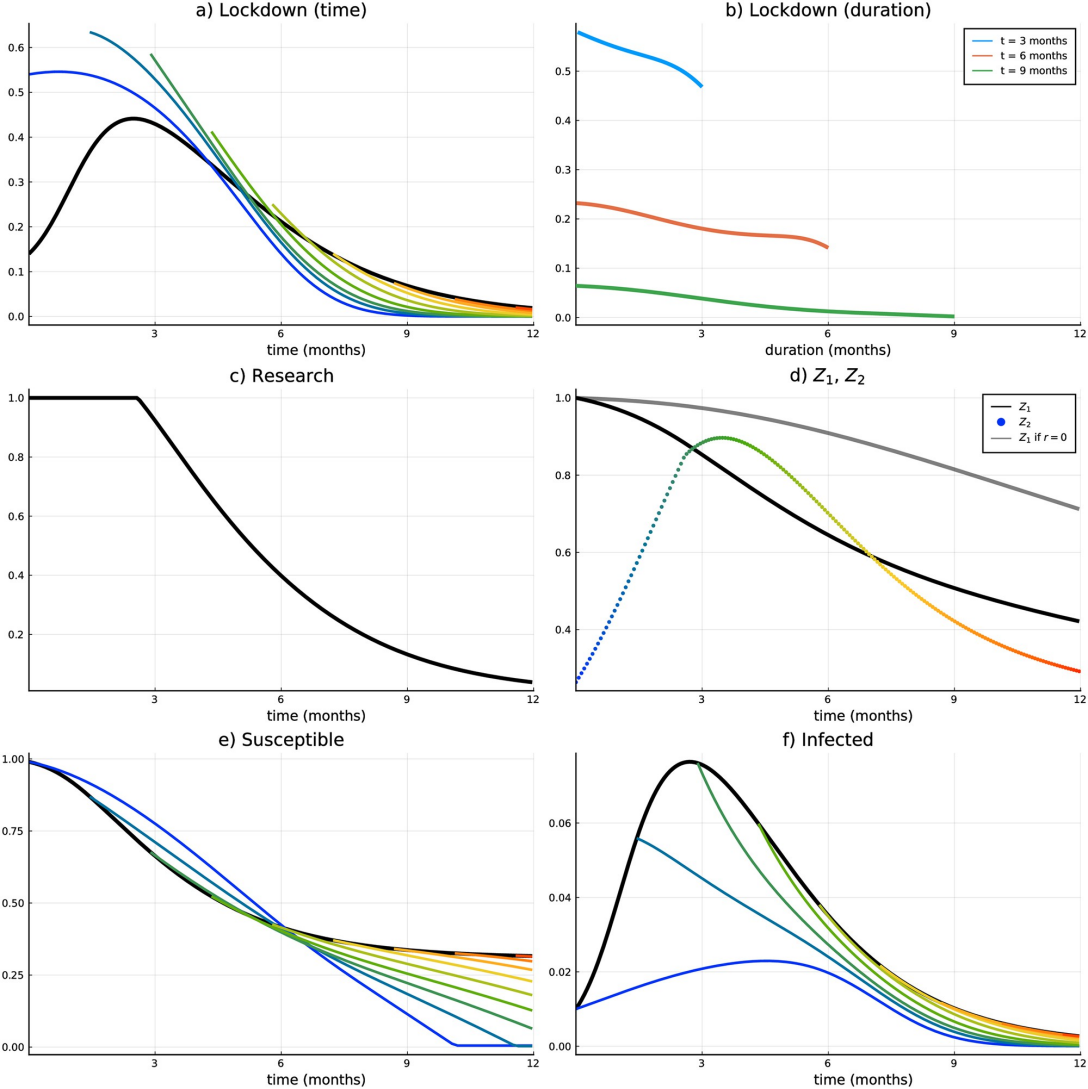
Scenario 1 (Course of the pandemic). (a) Lockdown intensity over time, (b) Lockdown intensity along duration, (c) Research effort over time, (d) Probability that *τ* has not set in yet, (e) Susceptibles, (f) Infected.

Fig 4 illustrates the most important variables along the course of the pandemic. Black lines in all subplots denote variables that correspond to the period before the vaccine has been approved (Stage 1). Colored lines and dots to variables thereafter. The grey line in panel (d) corresponds to the probability that a vaccine gets approved after *t* without an additional research boost at any time, i.e., if *r*_1_(*t*) = 0 for all *t*. All variables and values are plotted for the first year of the pandemic. Thereafter the solutions follow trajectories, which can be intuitively expected from the development within the first year (which means the pandemic ends, i.e., infections converge to zero, all people are getting the vaccine, lockdown is relaxed).

We start the discussion with the lockdown intensity plotted in panel (a). If a vaccine has not been approved, the intensity follows the course of the epidemic, i.e., the number of infected (plotted in panel (d)). This is reasonable in our modeling context since the number of deaths directly depends on the number of infected at *t* (see the definition of COVID-19 related death rate Eq (2)), and goes along with all recent papers on COVID-19 which assume lockdown intensity as a control variable. At the time the vaccine is approved, however, the lockdown is intensified if the approval happens in the first seven months of the pandemic. If the approval happens later on, the lockdown intensity remains approximately at the same level. After the upward jump, the lockdown intensity is adapted again continuously and relaxed earlier compared to the case without a vaccine (since the vaccine enables susceptibles to surpass the infected compartment). It is also obvious that for an early vaccine approval the consequent period with more intense lockdown measures is quite long. The number of infected is rather high and it takes time for the vaccination campaign (which is relatively slow at the beginning) to unfold its effect. For a late vaccine approval (after more than seven months) the lockdown jumps only marginally, but also ends earlier. Referring back to Theorem 2 an early approval corresponds to the first case of the theorem, while a late approval corresponds to the second or third case.

A different illustration of the lockdown intensity only after the vaccine approval is plotted in panel (b). The three colored lines represent the lockdown intensity over the time that has passed since the vaccine approval (i.e., duration of Stage 2, *d* = *t* − *τ*). For instance, the blue line shows the lockdown intensity that is implemented at three months across all possible approvals since the beginning of the pandemic (*d* ∈ [0, 3]). Thus, the lockdown value of the blue line with duration zero means the lockdown value of Stage 2 at *t* = 3 for *τ* = 3. The red and the green lines show the same for six and nine months respectively. From that figure, it becomes evident that (for the majority of points in time) the lockdown intensity is decreasing both in duration and in time. This can be followed from the strict decrease of the colored lines and from the fact that the lines which correspond to higher *t* start lower and never intersect with one another.

Research boost during Stage 1 is shown in panel (c) and will be discussed together with panel (d), which illustrates the probability that the vaccine will be approved after *t* (black line: optimal research efforts, grey line: zero research boost). The colored dotted line in panel (d) represents the probability density that the switch happens at *t*, technically that is the initial value of the auxiliary state variable *Z*_2_(*t*, *t*) (see S1 Appendix for details). Starting with the grey line in panel (d), we see that without additional research effort the probability that a vaccine is available on the market is only about 30% after one year. With the optimal additional research effort this chance increases and is twice as high (about 60%). For almost the whole first three months these additional efforts are at the maximum level in order to increase the probability density of the approval (colored dotted line in panel (d)). After that, additional efforts are decreasing, which is due to the fact that the peak of the pandemic has been passed (see number of infected in panel (f)) and the pandemic starts to decelerate. With a short time lag (which is due to increasing *p*(*t*)) the probability density for the vaccine approval (colored dotted line) also starts to decrease. For *t* approaching + ∞ the research boost, as well as the probability that the vaccine is approved after *t* and the probability density of the approval are converging towards zero. Please note again, that the approval rate and the corresponding probabilities cannot be calibrated at all and are furthermore very specific to the country of interest. As already mentioned in the introduction, China and Russia were the first countries to approve a (vector) vaccination already in summer 2020. The US and the EU approved two mRNA vaccines in December 2020. Moreover, the date of approval does not directly indicate the extent of the availability of vaccine shots (represented by $\alpha (\hat{t})$ in our model), which can be quite diverse across countries too.

**Fig 5:**
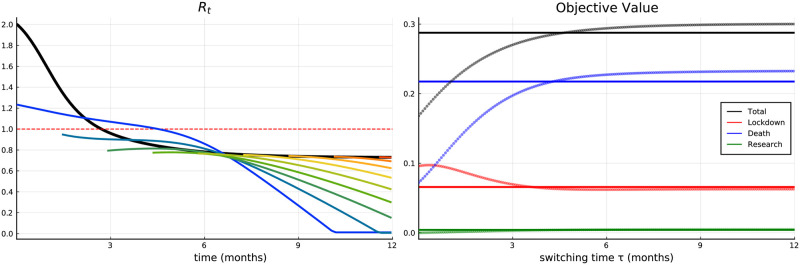
Scenario 1. Effective reproduction number over time (left) and decomposition of the objective value (right).

The left panel of Fig 5 strengthens the intuition of the qualitative shape of the lockdown intensity. It shows the effective reproduction number before and after vaccine approval over time, which can be derived by (see e.g., [25])

$$\begin{array}{c} R_{t,i}\left(t\right)=\frac{\beta \left(\ell_{i}\right)S_{i}/N_{i}}{\gamma },\;i=1,2. \end{array}$$ (25)

*R*_*t*_ equals the rate that one infected transmits the virus to one susceptible over the average duration of infectivity. In other words, it can also be interpreted as the average number of susceptibles that is infected by one infected individual. Therefore 1.0 denotes the important threshold, above which an epidemic shows dreaded exponential growth. The black line of Fig 5 (corresponding to Stage 1) starts from a considerably high value about 2.0, decreases steadily and drops below 1.0 shortly after the peak of the infection numbers. A stronger reduction is simply too expensive in terms of balancing lockdown and health economic costs (i.e., costs of lost lives). The situation changes, if the vaccine has already been approved (colored lines), i.e., during Stage 2 of our model. Due to the disruptive upward jump of the lockdown intensity, also the effective reproduction number jumps to a value around the critical threshold 1.0. In most cases, when the vaccine is approved after the first month of the pandemic, *R*_*t*_ directly drops down below 1.0. Again, this results from the more intensive lockdown paying off in these cases, since susceptibles can move directly to the *V* compartment. The consequent reduction of the *S* compartment is very fast, which also implies a decrease in the effective reproduction rate (faster decrease of the colored lines in Fig 5) and enables the government to relax the lockdown earlier.

The right panel of Fig 5 plots the total aggregated objective value (and a decomposition into its sub-parts), which realizes for different approval dates of the vaccine, i.e.,

$$\begin{array}{c} \int_{0}^{t}\;e^{-\rho s}g_{1}\left(I^{*}\left(s\right),\ell^{*}\left(s\right),r^{*}\left(s\right)\right)\;\;ds+\int_{t}^{+\infty }\;e^{-\rho s}g_{2}\left(I^{*}\left(s\right),\ell^{*}\left(s\right)\right)\;\;ds \end{array}$$ (26)

where the * denotes the optimality of control and state variables. Consider an approval at *τ* = 3 months. The corresponding objective value can be read at the value of the dotted black line at *t* = 3. The solid black line, on the other hand, denotes the expected value of the objective values described above, which is the objective function of our original problem Eqs (17)–(19), i.e.,

$$\begin{array}{c} E_{\tau }[\int_{0}^{\tau }\;e^{-\rho t}g_{1}\left(I^{*}\left(t\right),\ell^{*}\left(t\right),r^{*}\left(t\right)\right)\;\;dt+\int_{\tau }^{+\infty }\;e^{-\rho t}g_{2}\left(I^{*}\left(t\right),\ell^{*}\left(t\right)\right)\;\;dt]. \end{array}$$ (27)

Trivially this expected value is constant over *t*. The blue, red, and green lines, dotted and solid, can be interpreted analogously and represent the decomposition of the objective value into lockdown costs, health economic costs, and research costs (the three corresponding curves and dots sum up to the black ones). The total and health economics costs increase over time, i.e., an early vaccine reduces deaths resulting in lower costs. For early vaccine approvals, the corresponding costs are considerably lower than the expected value, but exceed it shortly before *t* = 5 months. On the other hand the economic costs resulting from the lockdown (red) show a different picture. The upward jump in lockdown intensity at the time of approval (for early vaccine approvals) implies decreasing total economic costs for later approvals. For an early vaccine approval the lockdown costs are higher than on average. However, for a vaccine approval after about four months the relation reverses, since the upward jump at the time of approval gets smaller and the period where the lockdown in Stage 2 is more intense than it would have been in Stage 1 gets shorter.

Research efforts are considerably cheap compared to all other costs. This goes along with empirical evidence. Basically all countries devoted as many financial resources as possible to support research. Qualitatively, they are analogous to total and health economic costs. All in all, health economic costs (blue curve) dominate lockdown (red curve) and research costs (green curve), both qualitatively and in absolute terms. This seems to be realistic considering reality. Being aware of the enormous costs of lockdown measures, countries all over the world did everything to keep deaths on a low level and agreed on huge financial supports of the economy. It is at hand that these results are sensitive concerning the (monetary) value of lost lives (measured in GDP per capita, see e.g., [26–29], and others). However, governments (and also researchers) face the same dilemma whenever health economic questions are considered (e.g., support research on medications, decisions on the payment of expensive treatment, decisions concerning the extension or reduction of the health system).

Numerical exercises for a broad range of parameters showed the qualitative robustness of the results.

#### Scenario 2: Vaccination within one month

Within this scenario, the same parameters as in Scenario 1 are used, except the parameters concerning the availability of the vaccines (see Eq (9)). We assume that all involved parameters (i.e., *α*_1_, *α*_2_, *α*_3_) are increased proportionally (this implies that the qualitative shape of *α*(*t*) remains unchanged) such that the whole population can be vaccinated within about one month (instead of 10 as in Scenario 1) after the approval. This is unrealistic and artificial. Israel was able to vaccinate 60 − 70% of the inoculable population within three months (see Fig 1) and is therefore still slower if compared to this scenario. However, it is useful in order to intensify the understanding of the model.

**Fig 6:**
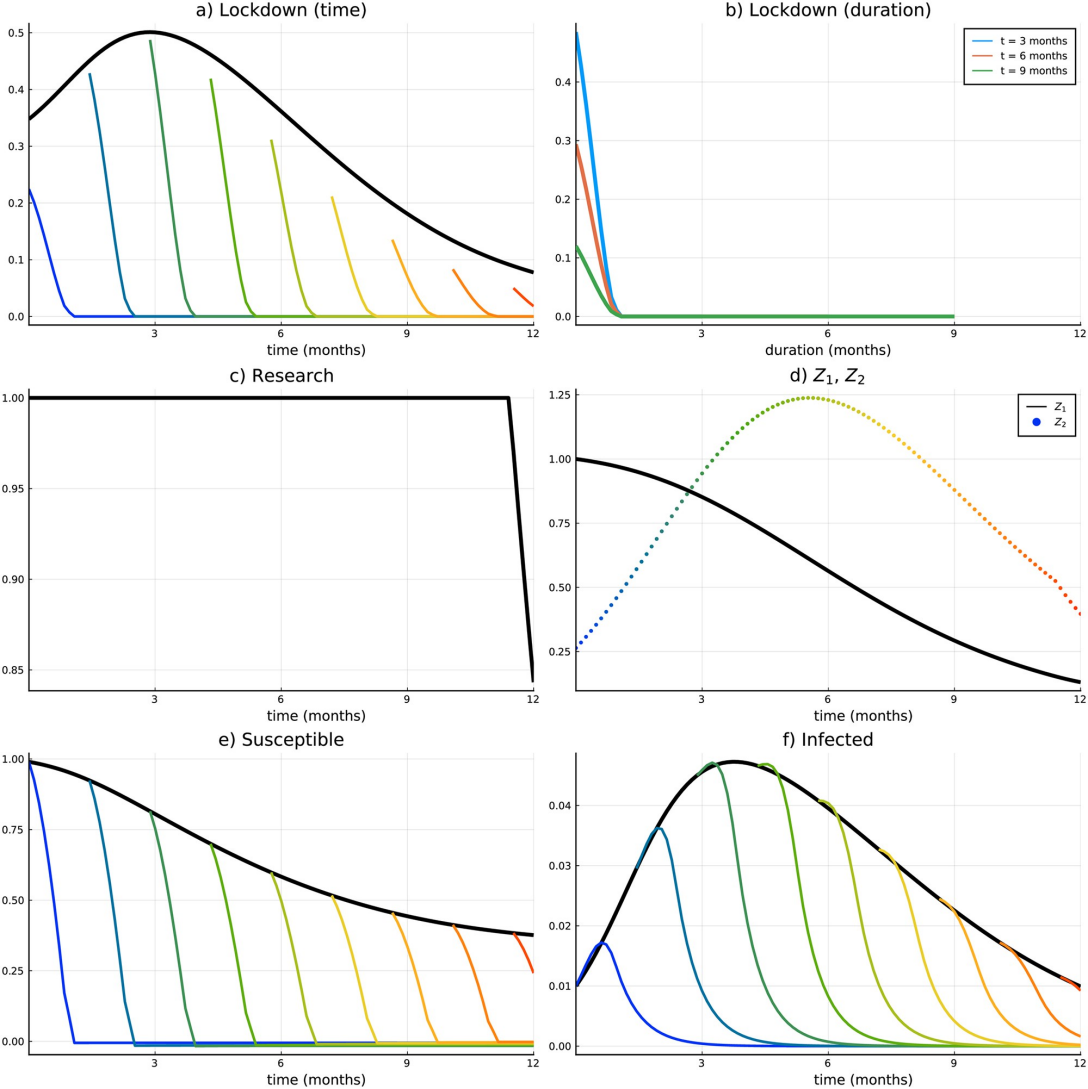
Scenario 2 (Course of the pandemic). (a) Lockdown intensity over time, (b) Lockdown intensity along duration, (c) Research effort over time, (d) Probability that *τ* has not set in yet, (e) Susceptibles, (f) Infected.

Fig 6 provides the same set of plots for Scenario 2 as Fig 4 for Scenario 1. I.e., lockdown intensity over time and along duration (panels (a) and (b)), additional research effort over time (panel (c)), probability that the approval happens after *t* and probability density of vaccine approval (panel (d)), susceptibles and infected (panels (e) and (f)).

The high speed of the vaccination campaign has a strong effect on the lockdown intensity at the time of the vaccination approval, as at that very instant it is optimal to immediately (and disruptively) reduce the lockdown measures. In Scenario 1 it was optimal to intensify the lockdown at the vaccine approval, to keep more people in the susceptible compartment and to move them directly to the *V* compartment instead of letting them undergo the COVID-19 infection process. This effect is undermined by the vaccination speed, since vaccinations work quicker than the infections in this scenario. Of course, the lockdown is not ended immediately, but decreases (continuously) to zero rapidly after the jump at the switch. This corresponds to the third case of Theorem 2, which means that the shadow price of a person getting infected at the switch (Stage 2) is lower than that without the switch (Stage 1) times the density for it.

The drastic continuous decrease of the lockdown after the vaccine approval in Stage 2 gets obvious in panel (b), showing the lockdown intensity along the duration. As in Scenario 1, the blue line corresponds to the lockdown over time if the vaccine is approved within three months, the red one for approval within six months, and the green one for approval within nine months. In all three cases, we see that the lockdown is ended about one month after the vaccine approval, irrespective of the lockdown intensity at the switch. That means that the lockdown should be ended completely only when almost all people have received the vaccination.

Panel (c) illustrates the research boost in Stage 1. Compared to Scenario 1, it is now at the maximum for almost one year (until the susceptibles are about 40% of the population). This is a clear consequence of a cost-benefit analysis of the corresponding effects. In Scenario 1 the population is vaccinated within about 10 months, which means that also after the R&D breakthrough considerably high lockdown costs arise. In the current scenario the vaccination administration is so successful that the lockdown can be relaxed shortly after the approval and completely dismissed soon after. Therefore, this implies much lower lockdown costs, and thus it is optimal to allocate as many financial resources as possible to increase the R&D success rate and consequently decrease the expected costs of the pandemic.

The probability to remain in Stage 1 and the probability density for the vaccine approval (both plotted in panel (d)) are changed according to the optimal path of the research efforts of panel (c). The black line (probability to stay in Stage 1) is lower than that of Scenario 1, while the colored one (probability density for approval) is higher.

The susceptibles (panel (e)) and infected (panel (f)) complete the picture for this scenario. Unsurprisingly, the susceptible compartment goes to zero within one month. The speed of the vaccination campaign implies that the pandemic will be ended soon after the vaccine approval. Therefore the government can afford higher infection numbers (compared to Stage 1) for a short time interval, which are due to the downward jump and the sharp decrease of the lockdown intensity.

### Anticipative versus non-anticipative behavior

Within this subsection, we compare the optimal solution of Scenario 1 (i.e., assuming a realistic speed of the vaccination campaign) to the case of a myopic government who does not anticipate a possible research breakthrough and the implied vaccine approval. This government just behaves as only Stage 1 would exist. If the vaccine is approved, the government (being surprised) immediately updates its decision and behaves according to the usual Stage 2 (as defined by Eq (19)).

**Fig 7:**
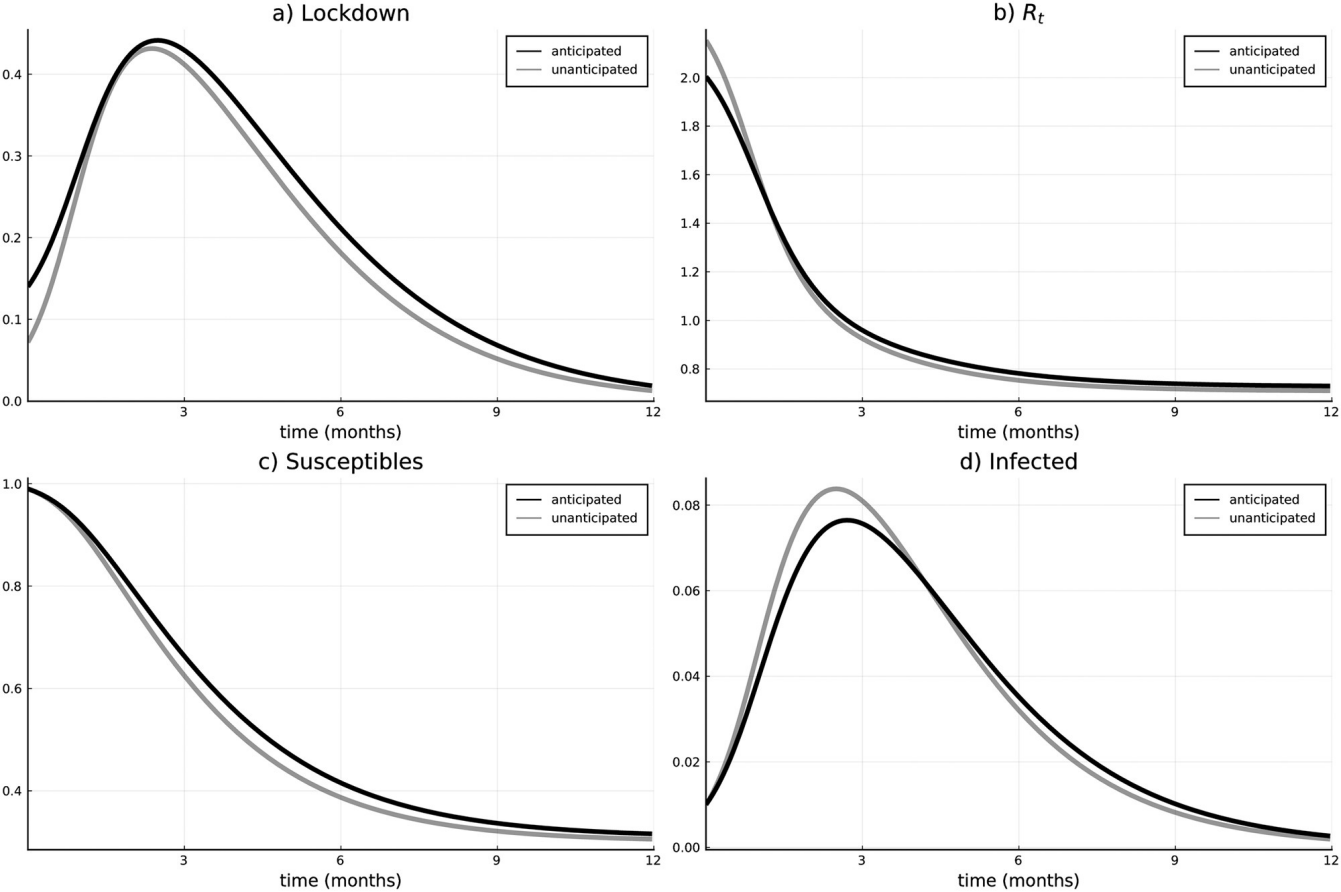
Anticipation versus non-anticipation. (a) Lockdown intensity in the anticipated and non-anticipated case, (b) Effective reproduction number in the anticipated and non-anticipated case, (c) Susceptibles, (d) Infected.

Fig 7 compares some key results of Scenario 1 with those of a myopic government. Panel (a) plots the lockdown intensity, panel (b) the effective reproduction number, panel (c) and (d) susceptibles and infected over time. All panels focus on Stage 1 since the behavior in Stage 2 is based on the same model and would overload the figure with too many lines.

The optimal lockdown intensity (panel (a)) turns out to be more restrictive if the arrival of a vaccine is anticipated. This results from the analogous effect, which lets the lockdown jump upwards at the time of vaccine approval in Scenario 1. I.e., if a vaccination can be expected, it is better to keep more people in the susceptible compartment so they can avoid the disease by getting the vaccine (if it becomes available). At the beginning of the pandemic, the lockdown intensity of the myopic case is only half of that of Scenario 1. Afterward the difference gets close to zero at the sharp increase before the peak of the pandemic (around *t* ≈ 1.5 months), finally, the difference increases again after the peak. Optimal lockdown intensities become more similar during the sharp increase since within this period the main driver of the lockdown intensity is not the current number of infected, but the dynamic nature of the pandemic (i.e., the *SI*-term in the dynamics). This implies that the lockdown is intensified in order to diminish this snowball effect, i.e., reducing high infection numbers (and therefore high costs due to lost lives) in the future. After that peak, the lockdown is again more intense in case of anticipation, but the difference is not as big as at the start of the pandemic, i.e., about 10%. Naturally, the difference tends to zero as the pandemic reaches its end.

The effective reproduction rate (panel (b)) is lower at the beginning of the pandemic for the anticipated case due to a more restrictive lockdown. This is due to the definition of *R*_*t*,*i*_(*t*) (see Eq (25)), where a strict lockdown (entering the nominator) decreases the fraction. However, already before the end of the first 1.5 months the relation switches and the unanticipated case has a lower effective reproduction number (together with a higher level of currently infected). This is due to the closeness of the lockdown intensities and due to the difference in the number of susceptibles. The latter effect dominates the effect of *φ*(*I*_*i*_) in the denominator (which increases the fraction).

Panels (c) and (d) mirror the trajectories of the lockdown intensity and effective reproduction rate. Initially, infections are higher in the unanticipated case due to the intensity of the lockdown, but they decrease after the peak of the pandemic. The number of susceptibles in the non-anticipated case does not catch up with that of the anticipated case, since the effective reproduction number gets lower when the number of infections already decreases. The intuition behind this is similar to the one given in the subsection presenting Scenario 1. Expecting a vaccine leads to the government trying to keep people healthy and to enable them to take the vaccine before they suffer the disease. The non-anticipative case causes more lost lives at the peak of the pandemic. If no vaccine becomes available, that might be the optimal solution. However, if a vaccine arrives, these additional lives are lost unnecessarily.

Several model runs with different parameters concerning the switching rate show qualitatively analogous results. However, the scale of the difference turns out to be sensitive. Especially, when a vaccine can be expected to be approved earlier (i.e., downward shift of the *Z*_1_(*t*) trajectory) the difference is remarkably bigger.

### Robustness check

In the definition of the model and in the previous section we proposed some simple functional specifications. As a result, we were able to reveal the causes of the qualitative behavior without any disturbances implied by involved (though more realistic) functions. Certainly, one may argue different assumptions and more complex functional forms, which raise questions on the validity of our key messages.

For a robustness check, we therefore analyzed the model with (i) convex-concave lockdown costs, (ii) time-dependent lockdown costs, and (iii) a finite time horizon (with different lengths). We report the obtained results here below.

#### (i) Convex-concave lockdown costs

In the sections “The model” and “Numerical results”, (c.f., Eq (13)), we propose convex lockdown costs due to the interconnectedness of the economy and the assumption that lockdown interventions are undertaken in order of cost-effectiveness. Although we believe that this assumption is reasonably argued, there might exist circumstances where initially increasing marginal costs of lockdown stringency *ℓ*(*t*) (i.e., convex part of the cost function) are followed by decreasing ones, when going beyond a certain threshold (i.e., concave part of the cost function). The intuition is that for a low *ℓ*(*t*) it becomes more and more difficult to find substitute inputs. Beyond a certain point, however, the economy is so constrained by low production levels, that further closures would have a decreasing effect on the implied costs.

To verify the validity of our core results also under an S-shaped/ convex-concave formulation, we assumed the following alternative form of the lockdown costs

$$\begin{array}{c} c_{\ell }\left(\ell \left(t\right)\right)=\frac{w}{3}(1-\text{cos}(\pi \ell (t))). \end{array}$$ (28)

**Fig 8:**
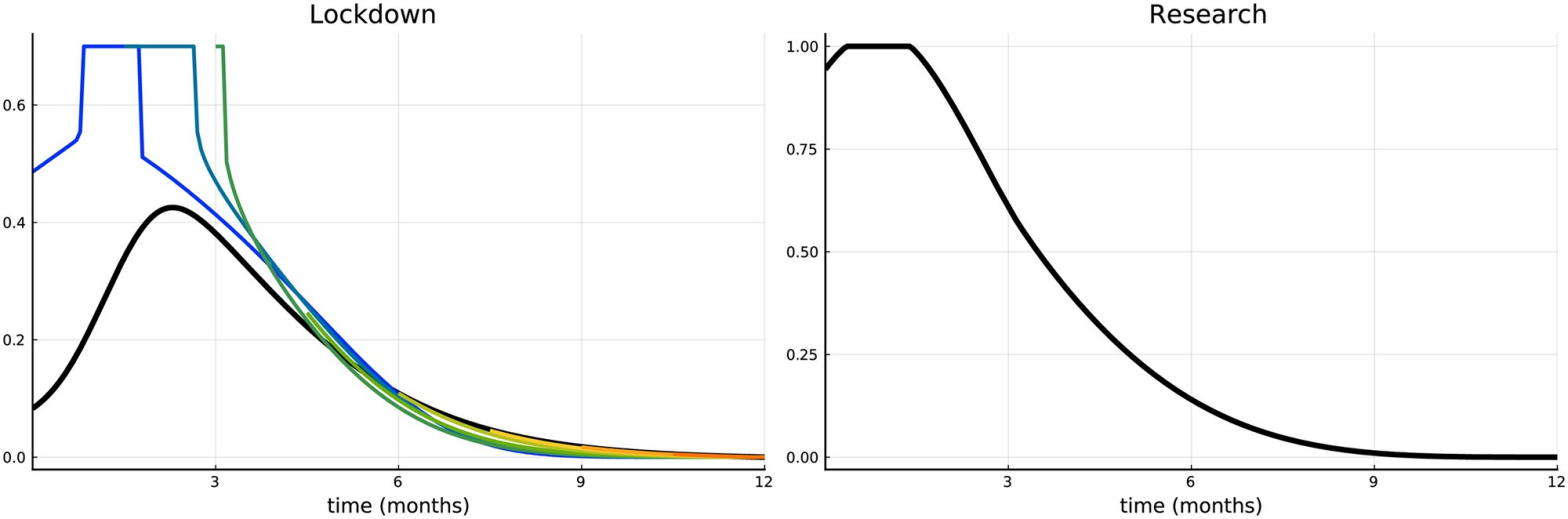
Convex-concave lockdown costs. Lockdown intensity over time (left) and research effort over time (right).

Function *c*_*ℓ*_(⋅) is convex for *ℓ*(*t*) ∈ [0, 0.5) and concave for $\ell \left(t\right)\in (0.5,\bar{L}]$. In Fig 8 we are plotting the optimal lockdown intensities and the research efforts over time for the vaccine availability of Scenario 1. In general, the qualitative nature of the optimal solution remains unchanged. At the time of vaccine approval, the lockdown has to be intensified for a short period, followed by an earlier relaxation afterward. However, due to the convex part of the cost function, the lockdown curve is not continuous: it jumps around the turning point of the cost function since it is not optimal to remain in the interior of the concave part of the cost function. Consider e.g., the blue curve. It is better to stay a little bit longer on a lower lockdown level and then immediately jump to $\bar{L}$. Also, the relaxation of the lockdown is initiated by a downward jump followed by a continuous change in the following (i.e., on the convex part of the cost function). Optimal research efforts are adapted accordingly.

Note that in general concave costs in an optimal control model frequently imply several problems like, e.g., chattering control (see e.g., [4]). However, in this example, a solution is possible since the cost function is convex only on the highest part and a direct jump to the maximum lockdown level solves the issue.

#### (ii) Time-depending lockdown costs

For the second part of the robustness check we relax the assumption of time-independent lockdown costs. In the beginning of the pandemic, firms could use reserves or inventories of inputs to partially absorb the supply problems. After some time, as reserves or inventories are exhausted, economic constraints become binding. Therefore, we introduce an additional factor to the cost function, which increases over time, i.e.,

$$\begin{array}{c} c_{\ell }\left(\ell \left(t\right)\right)=e^{3t}w\ell^{2}\left(t\right). \end{array}$$ (29)

**Fig 9:**
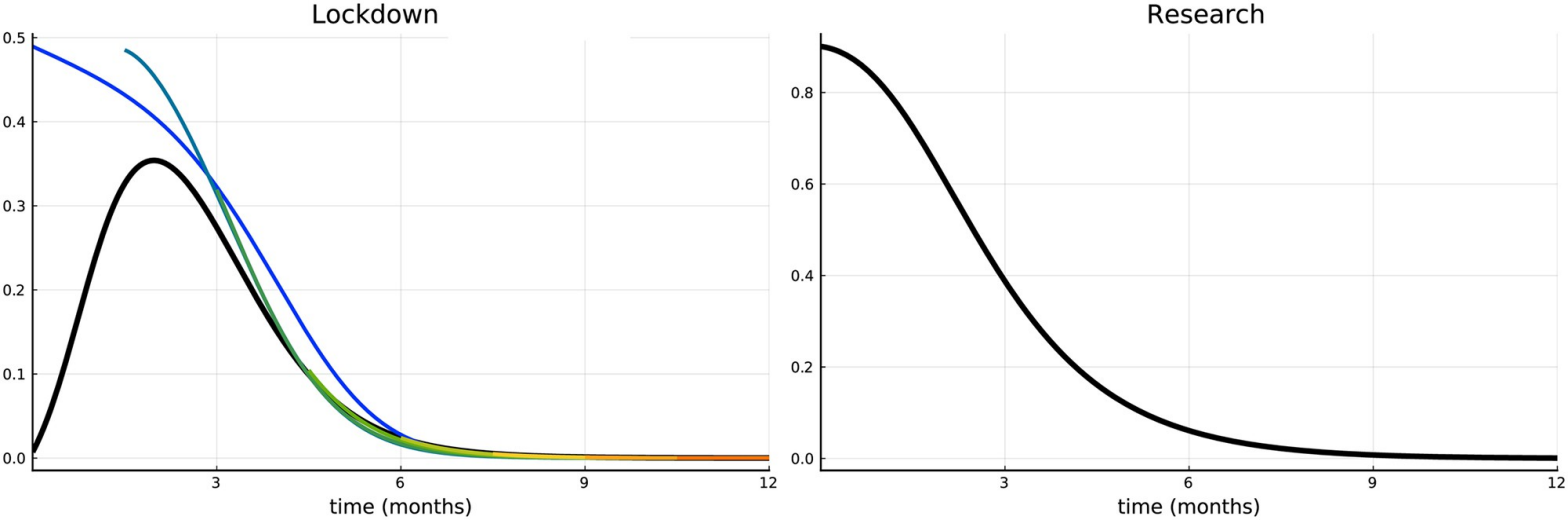
Time dependent lockdown costs. Lockdown intensity over time (left) and research effort over time (right).

For *t* = 0 costs are identical to our benchmark and after e.g., six months they are multiplied by a factor *e*^3⋅0.5^ ≈ 4.48 (the factor *e*^3*t*^ has been chosen for illustration proposes only and does not reflect realistic data). Increasing *t* makes lockdown more costly. Fig 9 plots the optimal lockdown intensity over time and research effort (with the rest of the parameters being equal to Scenario 1). Our core results still hold, but are slightly adapted. Increasing lockdown costs over time naturally implies that the lockdown will tend to be lower (compared to the benchmark). This, in particular, holds for the upward jump at the approval of a vaccine. While at early approvals the jump is evident, later on (about four months after) the discontinuity almost disappears, since during this time the lockdown has already become very expensive. The optimal research efforts also decreased considerably, which is a result of more people getting immunized through infection. Lower lockdown intensity implies more infections at the beginning, but a lower number of susceptibles and a higher number of recovered thereafter.

#### Effect of time horizon

As a third robustness check, we vary the time horizon. In our basic setup and in the “Numerical results” we have chosen an infinite time horizon (as used in e.g., [16]), which corresponds to socially optimal decisions (the cost of the entire duration of the pandemic are evaluated). However, in reality, democratically elected governments are in place until the next election. While several papers, therefore, assume a finite time horizon with a zero salvage value function (see e.g., [7], or [9]), we believe that a salvage value function is important. The government has the responsibility for the epidemic situation when elections take place (see e.g., [12, 14], or [30]).

For illustration, we assume that the decision-maker evaluates a proxy of the expected health costs of the infected individuals at the end of the time horizon *T*. This is governed by the following salvage value function

$$\begin{array}{c} S\left(I\left(T\right),T\right)=e^{-\rho T}c_{h}\left(\bar{I}\left(T\right)\right)\;\text{where}\;\bar{I}\left(T\right)≔I\left(T\right)\int_{T}^{\infty }e^{-\gamma t}\;\;dt=\frac{e^{-\gamma T}}{\gamma }I\left(T\right). \end{array}$$ (30)

Individuals are leaving the *I*-compartment with rate *γ*. To account for the future time spent in the *I*-state by the final *I*(*T*) infected individuals, $\bar{I}\left(T\right)$ captures the approximate duration-adjusted infected. Deriving the health economic cost of this number is a proxy for the implied costs.

**Fig 10:**
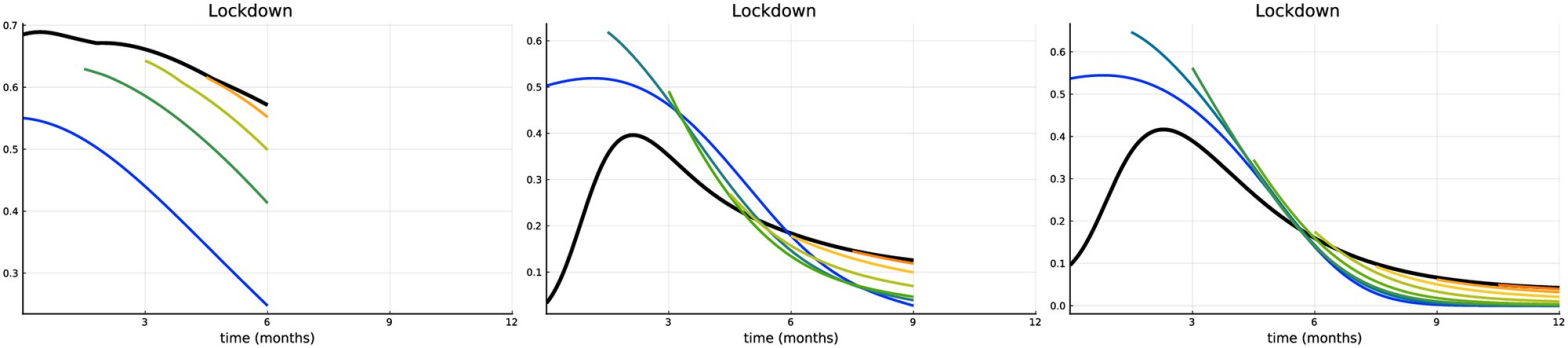
(iii) Finite time horizon. Lockdown intensity with time horizon of six months (left), nine months (middle) and one year (right).

Fig 10 plots the optimal lockdown intensity time horizons of six months (left panel), nine months (middle panel), and one year (right panel). In the case of a time horizon of one year (or longer) the results are practically identical to those of the infinite time horizon (see Scenario 1) since after one year the number of susceptibles is very low in our setting. Reducing the time horizon to nine months is enough to see a slight change in the solution. Decreasing the time horizon further finally reveals a drastic change of the optimal policy. The left panel shows the situation for six months. The lockdown does not abruptly increase but instead drops down at the vaccine approval, because it is not possible to profit from the following earlier relaxation during the remaining short time horizon. Moreover, it is not possible to significantly reduce the pandemic burden to a low level within this time frame. Therefore it is more efficient to save costs in terms of the lockdown.

In a nutshell, the difference between the results of the finite time and the infinite time horizon is more pronounced the shorter the time horizon. I.e., policy-makers tend to deviate from the socially optimal solution the sooner their legislation period ends.

## Conclusions

### Overview

The current model extends the existing epidemiological models by specifying how a vaccine and its arrival are included in the optimization process. Using our approach we are able to overcome the following two crucial limitations: the *(un)known arrival/approval time* of the vaccine and the *continuation of the COVID-19 pandemic* during a vaccination campaign until enough people are immunized.

The formal analysis (see Theorem 2) and the numerical results show that the lockdown intensity (which is assumed to vary continuously in our model) jumps at the approval of the vaccine. In a realistic scenario, where the whole population will be vaccinated within 10 months, the lockdown is even intensified for a short period followed by an earlier relaxation due to the reduction of the susceptibles. This is consistent with the result of [19], which is obtained using a different model setup and focus. However, it contrasts with other papers (e.g., [17]), which claim that lockdown and vaccination are always substitutes. The main driver behind this upward jump is that more lives can be saved, if more people are kept susceptible (by intensifying the lockdown) and can move directly to the vaccinated compartment. In an artificial scenario, where the vaccination campaign works very fast, i.e., vaccination of the population within one month, the adaptation of the lockdown at the approval date is reversed. The effect of the vaccine dominates the effect discussed above, which saves both lockdown costs, by relaxing the lockdown intensity, and lives, by the rapid reduction of the susceptibles.

A comparison of the optimal solution with the myopic case, where the government does not include the potential approval of an effective vaccine at all, shows that the expectation of a vaccine leads to a stricter lockdown policy. The explanation is similar to the jump at the vaccine approval date: A vaccine enables susceptibles to surpass the infection. Thus a “well-ordered” flow from the susceptibles to the infected, keeping the number of deaths at an acceptable level, becomes unnecessary at the time the vaccine becomes available.

The section “Robustness check” illustrates variations of the assumptions concerning the lockdown costs (convex-concave, time-dependent) and the time horizon. Our key findings turn out to be robust w.r.t. changes in the lockdown costs and for long finite time horizon. For a short time horizon, the qualitative solution structure changes. It is no longer optimal to increase but to decrease the lockdown at the vaccine approval.

By additional numerical calculations (not included in this paper) we presume that the qualitative results of our analysis are also robust against other parameter changes. In all variations, the key message remains that the vaccine availability shapes the structural characteristics of the solution. This is shown by two different scenarios.

### Limitations

The paper suffers from a number of limitations, which propose further extensions of the model.

In Stage 2 of our model, it is assumed that the vaccination is 100% effective. While the mRNA vaccines of Biontech/Pfizer and Moderna are relatively close with more the 94 − 95% effectiveness, other vaccines are less effective. Moreover, this effectiveness has been tested by large studies a couple of months before the corresponding approval date. In the meantime the virus has mutated, and this might have also changed the vaccine effectiveness. For the “*α*” and “*δ*”-variants (earlier called “British variant” or B.1.1.7 and “Indian variant” or B.1.617), which have replaced the original strain of the virus in Europe, the currently approved vaccines seem to remain quite effective (at least in preventing a heavy course of the disease). However, virologists expect also “escape mutations” to arise in the near future (the Omicron-variant took over in late 2021/early 2022). As a consequence, it seems to be realistic that a COVID-19 vaccine booster (adapted to new variants) will be necessary on a yearly basis, as it is common for the influenza virus. Including this fact into our model means adding more compartments and flows between them. This will certainly add new effects to the optimal solution. To assess their impact in further steps, it is crucial to understand the base effect within the current model first.

Another critical assumption of the current model is that the effectiveness of the lockdown remains constant over time, independently of its duration and intensity. Observing that the resentment in the population is growing since fall 2020 in many countries this assumption seems to be problematic. This so-called “lockdown fatigue” effect is thoroughly addressed in [13, 14]. The analysis shows that the shape of the optimal lockdown over time need not be hump-shaped, but can have several peaks. Even several lockdown periods are possible. However, these papers assume a finite time horizon without vaccination, which corresponds to the myopic scenario in our paper. Therefore, the models are not directly comparable to our setup, and our model should be enriched with the lockdown fatigue effect to investigate this important question.

Finally, note that our paper abstracts from the possibility of testing and tracing (including quarantine) strategies. This is, of course, another important modeling option that might make the results more realistic and comparable to the real world. And indeed, the adaptation could straightforwardly be done by adding additional compartments (for identified people) and a parameter or a control variable (for testing efforts). This route has been followed in [30] again without vaccination. The analysis shows that testing has a strong effect on the pandemic if tracing is efficient. In the case of inefficient tracing, the result is basically equal to a solution without any testing. We have chosen to remain in our setup, since the focus of this paper is clearly different and allows a straightforward identification of the additional channel by the stochastic arrival of the vaccine.

## Supporting information

S1 Appendix Age-structured formulation of the model. (ZIP)
